# Inhibition of experimental myopia by a dopamine agonist: different effectiveness between form deprivation and hyperopic defocus in guinea pigs

**Published:** 2011-10-31

**Authors:** Feng Dong, Zhina Zhi, Miaozhen Pan, Ruozhong Xie, Xiaoyi Qin, Runxia Lu, Xinjie Mao, Jiang-Fan Chen, Mark D.P. Willcox, Jia Qu, Xiangtian Zhou

**Affiliations:** 1 School of Optometry and Ophthalmology and Eye Hospital, Wenzhou Medical College, Wenzhou, Zhejiang, China; 2 State Key Laboratory Cultivation Base and Key Laboratory of Vision Science, Ministry of Health P.R. China and Zhejiang Provincial Key Laboratory of Ophthalmology and Optometry, Wenzhou, Zhejiang, China; 3 Department of Neurology, Boston University School of Medicine, Boston, MA; 4 School of Optometry and Vision Science, The University of New South Wales, Sydney, NSW, Australia

## Abstract

**Purpose:**

The dopamine (DA) system in the retina is critical to normal visual development as lack of retinal DA signaling may contribute to myopic development. The involvement of DA in myopic development is complex and may be different between form deprivation and hyperopic defocus. This study evaluated effects of a non-selective DA receptor agonist, apomorphine (APO) on refractive development in guinea pigs treated with form deprivation or hyperopic defocus.

**Methods:**

APO was subconjunctivally injected daily for 11 days in form-deprived (0.025 to 2.5 ng/µl) and defocused (0.025 to 250 ng/µl) eyes. Changes in ocular biometry and retinal concentration of DA and its metabolites (DOPAC) were measured in the 2 animal models to assess the level of DA involvement in each of the models (the less the change, the lower the involvement).

**Results:**

Similar myopic degree was induced in both the deprived and defocused eyes (−4.06 D versus −3.64 D) at 11 days of the experiment. DA and DOPAC levels were reduced in the deprived eyes but did not change significantly in the defocused eyes compared to the fellow and normal control eyes. A subconjunctival injection of APO daily for 11 days at concentrations ranged from 0.025 to 2.5 ng/µl inhibited form deprivation myopia in a concentration-dependent manner. By contrast, the APO treatment ranged from 0.025 to 250 ng/µl did not effectively inhibit the defocus-induced myopia and the associated axial elongation.

**Conclusions:**

DA signaling may play a more critical role in form deprivation myopia than in defocus-induced myopia, raising a question whether the mechanisms of DA signaling are different under these two types of experimental myopia.

## Introduction

Dopamine (DA) signaling in the retina is believed to be critical during the development of experimental myopia [1-6]. Retinal DA is released exclusively from amacrine or interplexiform cells and its release increases during daytime or illumination but declines in darkness [7,8]. Myopia can be induced experimentally either by form deprivation or hyperopic defocus of the retinal image. These 2 visual manipulations cause axial elongation and choroid thinning of the eye, resulting in axial myopia [2,9,10]. Similar changes in gene and protein expression of some growth factors, such as egr-1 (early growth response protein 1; ZENK), glucagon, transforming growth factor (TGF) and crystallins also occur in the retina of the eyes treated with either form deprivation or hyperopic defocus [11-15]. Furthermore, the refractive development in these 2 models can be similarly modified by controlling the axial lengthening of the eye with dopaminergic agonists and muscarinic acetylcholine receptor (mAChR) antagonists [5,16,17], suggesting that both the DA and cholinergic systems are involved in refractive development of the eye.

Some mAChR antagonists including atropine and pirenzepine have been used to prevent or inhibit the development of myopia for decades in both clinical and experimental settings [17,19-24]. However, the long-term effect of mAChR antagonists on inhibition of myopia is not fully determined [25,26] and side-effects caused by mAChR antagonists are unacceptable for some patients. Therefore, these agents are not ideal as long-term medications for prevention of myopic development [23,25]. DA appears to function similarly to mAChR antagonists in biologic control of experimental myopia [11,16,18,27,28]. DA and its agonists have been used routinely to treat Parkinson disease [29] and therefore would be acceptable clinically if they were proved to be effective in the treatment of myopia.

Activation of DA receptors by local administration (intravitreal, subconjunctival or topical) of dopamine, levodopa (a precursor of dopamine) or apomorphine (APO, a non-selective DA receptor agonist) can inhibit form deprivation myopia (FDM) in guinea pigs, rabbits and rhesus monkeys [5,28,30,31], while APO and quipirole (a D2 receptor agonist) inhibit both form deprivation and defocus-induced myopia in chickens [16,32]. Biometric changes in these myopic eyes mainly manifest as axial elongation of the eye and transient thinning of the choroid [5,16,32-37]. However, chicken eyes frequently exposed to flickering (a dopamine synthesis stimulator as shown by Umino et al. [38] and Dong & McReynolds [39]) do not develop form deprivation- or defocus-induced myopia [1,40]. Furthermore, D2 receptor antagonist, sulpiride can enhance FDM [41] and another D2 antagonist spiperone used together with APO can compromise the role of APO in inhibition of FDM [42]. These results indicate that retinal DA receptors are involved in dopaminergic control of ocular axial growth and the availability/or susceptibility of the DA receptors plays a crucial role in this dopaminergic effect. In contrast, depletion of retinal DA by 6-hydroxydopamine (6-OHDA, a neurotoxin) or reserpine inhibits both FDM and defocus-induced myopia in chickens [27,41,43,44]. As a neurotoxin, 6-OHDA may not only deplete the retinal DA but also damage other retinal cells and tissues, resulting in a non-dopamine specific retardation of the eye growth. Therefore, these seemingly paradoxical results with both activation and inactivation of DA signaling should be taken into account with the toxicity and specificity of the pharmacological agents used.

Although both FDM and defocus-induced myopia share some similarities in genetics, proteins and neurobiological activities, the level of involvement and biologic responses of the ocular-neurologic system appear different in these 2 models [17,45,46]. For instance, the levels of DA and its major metabolite, 3, 4-dihydroxyphenylacetic acid (DOPAC) are reduced in the retina of chickens and monkeys following form deprivation [27,47]. This is consistent with a decreased rate of retinal DA release in FDM [43] and the rapid recovery of retinal DA and DOPAC levels in the chicken eyes recovering from FDM [48]. On the other hand, reported levels of retinal DA during defocus-induced myopia are inconsistent in the literatures [41,49], possibly due to the difference in power of the negative lenses used. This hypothesis is evidenced with an increased sensitivity of the eye to the suppressive effect of APO on defocus-induced myopia when the negative-lens power is increased [10,41]. A more recent study shows that APO is more effective in control of defocus-induced myopia than in FDM in 8-day old chickens [16]. Atropine has a greater inhibitory effect than the combination of atropine and APO on FDM, but this is not the case for defocus-induced myopia [16]. Constant lighting, which breaks the diurnal cycle of DA levels in the retina can inhibit FDM, but does not affect development of defocus-induced myopia [50]. Finally, the growth of sclera in response to the negative lens wear is much more rapid than that to form deprivation [46]. All of these results indicate that mechanisms mediated by the dopaminergic system in control of axial growth of the eye are not exactly the same between form deprivation and hyperopic defocus in chicken models.

An understanding of DA involved in the development of myopia could help select potential medical treatment for refractive errors. At present, neurochemical mechanisms involved in myopia development have not been studied as extensively in mammals as in chickens although studies on monkeys and tree shrews have provided some results similar to those in chickens [51,52]. Guinea pigs are a promising alternative to chickens and other mammals for the study of experimental myopia [34,53-56]. They develop myopia more rapidly compared to monkeys [57-59] and have shown that local DA signaling plays a significant role in inhibition of form deprivation myopia [5,6,60].

In this study, we aimed to explore the possible different roles of DA signaling between the FDM and defocus-induced myopia using guinea pigs. Specifically, we investigated effects of FDM and defocus-induced myopia on retinal DA concentration and the associated metabolism. Furthermore, we determined the effect of local administration of the non-selective DA agonist APO on refraction and the associated biometric changes in FDM and defocus-induced myopia. A potency curve of different doses of APO used for guinea pigs was also established as compared to that from the chicken models.

## Methods

### Experimental design

The animal research in this study was approved by the Animal Care and Ethics Committee at Wenzhou Medical College (Wenzhou, China). Treatment and care of animals were conducted according to the ARVO Statement for the Use of Animals in Ophthalmic and Vision Research. One hundred and forty pigmented guinea pigs at age of 3 weeks were randomly assigned to FDM (a facemask worn monocularly) or defocus-induced myopia (a -4.00 D [diopter] lens worn monocularly) and control groups. The FDM groups were treated with 0.025 ng/µl APO (n=8), 0.25 ng/µl APO (n=7), 2.5 ng/µl APO (n=6), vehicle (0.1 mg/ml ascorbic acid, n=9), or FDM-only (n=6). The defocus-induced myopia groups were treated with 0.025 ng/µl APO (n=12), 0.25 ng/µl APO (n=13), 2.5 ng/µl APO (n=14), 25 ng/µl APO (n=8), 250 ng/µl APO (n=8), vehicle (0.1 mg/ml ascorbic acid, n=14), or defocus-only (n=17). The control groups were treated with 2.5 ng/µl APO (n=6), vehicle (0.1 mg/ml ascorbic acid, n=6), or no treatment (n=6). APO dissolved in 100 μl vehicle (or vehicle alone) was administered monocularly by subconjunctival injection into eyes of the designated groups. Ocular biometric parameters were measured in both eyes of individual animals before and at 11 days of treatment.

### Establishment of axial myopia by form deprivation and hyperopic defocus

FDM was achieved using a latex shield to cover one eye, as described previously [54]. For defocus-induced myopia, a latex-made facemask was held in place by a rubber-band around the head of animals, leaving both eyes, the nose, mouth and ears freely exposed. A - 4.00 D lens (Boston IV, diameter: 11.8 mm, optical zone: 11.0 mm, base curve: 12.0 mm; Xinshijie, Wenzhou, China) was glued onto a plastic lens frame. The lens frame was then attached to the facemask around one eye by a fabric hook-and-loop fastener (Velcro; Hongxin, Shenzhen, China) after the optical center of the lens was aligned with the pupil center. The lens was detached and cleaned on both sides with a water-wetted gauze at least once daily followed by re-attachment to the facemask. All the animals were maintained on a cycle of 12-h illumination (500 Lux) and 12-h darkness during the experimental period.

### Pharmacological manipulation

APO (Tocris, Glasgow, UK) solution at different concentrations was freshly prepared before each injection. The drug was dissolved in sterilized injection water with ascorbic acid added (0.1 mg/ml) as an antioxidant. The vehicle solution contained 0.1 mg/ml of ascorbic acid in sterilized injection water. Only one eye of each animal received the injection (deprived eyes in FDM groups; defocused eyes in defocus-induced myopia groups; randomized right or left eyes in other groups). Topical anesthesia was administered with 1 to 2 drops of 0.5% proparacaine hydrochloride (Alcon, Puurs, Belgium) after removal of the facemask or lens. A subconjunctival injection of 100 μl APO solution with a concentration from 0.025 ng/µl to 250 ng/µl APO (same volume for vehicle injection) was performed using a syringe with a 26-gauge needle once daily (at 9 AM) for 11 days. The injection site was just through the conjunctival reflection, 4 mm inferior to the lower corneal margin. The facemask or lens was placed back on the eye immediately after each injection. The entire injection period from removal to replacement of the MDF or lens was approximately 2 min.

### Measurements of vitreous APO concentration after subconjunctival injection

To confirm the concentration of APO in the injected eyes, 45 extra animals (2 eyes of each) were used to measure the vitreous concentration of APO using HPLC before (n=2) and at 0.5 (n=6), 1 (n=6), 6 (n=12), 12 (n=14), and 24 (n=15) h after the subconjunctival injection. The eyes were placed in liquid nitrogen for about 20 s and hemisected sagittally in an iced box. The vitreous body was gently harvested using an iced 1.5-ml Eppendorf (EP) tube, mixed with ascorbic acid (final concentration 1.0 mg/ml) and centrifuged at 1,250× g at 4 °C for 5 min. The supernatant (containing APO) was transferred to another EP tube and stored at −80 °C. All vitreous samples from the same time point were pooled into one collected sample which was analyzed 3 times to provide the mean value of APO. The analytical column was packed with a 5 µm Zorbax Eclipse XDB C18 (Agilent, Santa Clara, CA) at 30 °C with a 5 µm XDB C18 as a guard column (Agilent). The mobile phase ran a mixture (30:70, v/v) of methanol and a solution of 12 mmol/l sodium dihydrogen phosphate plus 1 mmol/l (w/v) EDTA adjusted to pH 3.00 with orthophosphoric acid at a flow rate of 1.0 ml/min. Wavelengths of excitation and emission were 276 nm and 460 nm respectively for both APO and boldine (internal standard, IS). Retention time for APO and IS were 4.4 min and 3.7 min, respectively.

### Biometric measurements

Biometric parameters (refraction, corneal curvature, and axial components of the eye) were measured by an optometrist with help from an animal care assistant during the light cycle (daytime) after removal of the facemask or lens. The optometrist was masked in regard to the treatment conditions for each animal.

Refraction was measured by retinoscopy after the pupil was completely dilated by topical administration of 1% cyclopentolate hydrochloride (Alcon, Fort worth, TX). The results of retinoscopy were recorded as the mean value of the horizontal and vertical meridians [54-56]. Corneal curvature was measured with a keratometer (OM-4; Topcon, Tokyo, Japan) modified by attachment of an +8 D lens onto the anterior surface of the keratometer. A group of stainless steel balls with diameters from 5.5 to 11.0 mm were measured by the modified keratometer. Three readings were recorded for each measurement to provide a mean result. The radius of corneal curvature was then deduced from the readings on the balls with known radii [54].

A-scan ultrasonagraph (Cinescan A/B, frequency: 11MHz; Clermont Ferrand, France) was used to measure axial components of the eye (lens thickness and vitreous length and axial length). The conducting velocity was 1,723.3 m/s for measurement of the lens thickness and 1,540 m/s for measurement of the vitreous length as described previously [56]. Each of the axial components was calculated as the mean of 10 repeated measurements.

### Measurements of levels of retinal DA and DOPAC

#### Sample preparation

Sixty-three extra animals (2 eyes of each) were used to measure retinal levels of DA and DOPAC under normal visual conditions (n=14) and at 11 days of form deprivation (n=26) or hyperopic defocus (n=23). After enucleation of the eye, the retina was dissected on an iced dish, weighed, homogenized in 150 µl of 1M HClO_4_ with 3, 4-dihydroxybenzylamine (DHBA; Fluka, Milwaukee, WI), centrifugated at 30,000× g for 30 min at 4 °C and finally the supernatant was collected and stored at −80 °C [7,61].

#### Chromatographic process

The analytical column was packed with a 5 µm Diamonsil C18 (4.6 mm×250 mm I.D.; Dikma, Shanghai, China) at 30 °C with a 5 µm XDB C18 as a guard column (4.6 mm×12.5 mm I.D.; Agilent). The mobile phase ran a mixture (83:17, v/v) of methanol and citrate buffer solution (0.08 M, pH 4.4; citric acid 50 mM,trisodium citrate 30 mM,octane 0.83 mM,EDTA 0.1 Mm) at a flow rate of 1.0 ml/min. The volume of analyzed samples for HPLC was 40 µl. Retention times for DA, DOPAC and internal standard DHBA were 8.4, 10.0, and 11.4 min, respectively. Peaks of the three agents were separated without any interfering peaks between them. DA (Sigma, Buchs, Switzerland) and DOPAC (Aldrich, Buchs, Switzerland) at five different concentrations (2.5, 5.0, 12.5,5.0,10.0, 25.0, and 100.0 ng/ml) were used to form a standard curve with DHBA (concentration: 30 ng/ml) as an internal standard for calibration of the drug concentration.

Immediately before analysis, perchloric acid was removed from the samples by precipitation with potassium citrate solution and centrifugation. The supernatant was passed through a 0.22 µm membrane (Millipore, Billerica, MA) and an aliquot of 40 µl was injected for HPLC analysis. The DA/DHBA and DOPAC/DHVA peak-area ratios (As/Ai) were plotted against the ratio of the corresponding concentrations (C). The concentrations of DA and DOPAC were expressed as ng/mg wet-weight retina [7,61].

### Statistics

Biometric results were compared between deprived/defocused eyes and their fellow eyes within the same group using a paired sample *t*-test, SPSS Version 12.0 (SPSS, Chicago, IL). These results were also compared between different time points in the same group by independent *t*-test and among different groups by one-way ANOVA with Bonferroni correction, SPSS Version 12.0. Both the intra-group and inter-group differences were defined as significant at p<0.05 and highly significant at p<0.01.

## Results

### Levels of retinal DA and DOPAC in different visual environments

**Figure 1:**
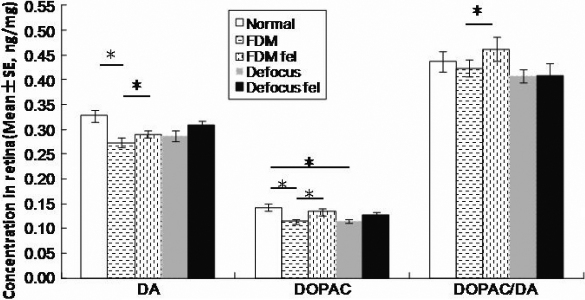
Retinal DA and DOPAC levels and the DOPAC/DA ratio in normal control, FDM-only and defocus-only groups. DA and DOPAC concentrations were determined in retinal extracts at day 11 of treatment (FDM: deprived eyes; FDM fel: fellow eyes to deprived eyes; defocus: defocused eyes; defocus fel: fellow eyes to defocused eyes). The levels of retinal DA, DOPAC, and the DOPAC/DA ratio were significantly lower in the deprived eyes compared to their fellow eyes (* p<0.05, paired sample *t*-test). The levels of DA and DOPAC in the deprived eyes were significantly lower than in the normal control eyes (*p<0.05, one-way ANOVA). In contrast, the defocus-only group showed similar levels of DA and its metabolites between eyes of the individual animals (p>0.05, one-way ANOVA). However, the defocused eyes showed a significant reduction in retinal DOPAC level but no significant changes in DA or DOPAC/DA levels when compared to the normal control eyes (*p<0.05, one-way ANOVA).

There was no significant difference in levels of retinal DA, DOPAC, and DOPAC/DA between eyes of individual animals in the normal control group (Table 1 and Figure 1), or between the right eyes of the normal control group and the fellow eyes in FDM-only and defocus-only groups. However, the levels of DA, DOPAC and DOPAC/DA in the deprived eyes were significantly lower than in the fellow eyes (FDM versus FDM fellow: 0.273 versus 0.292 ng/mg for DA, 0.114 versus 0.134 ng/mg for DOPCA, 0.424 versus 0.462 ng/mg for DOPAC/DA; p≤0.042, paired sample *t*-test) and the normal control eyes (FDM versus normal: 0.273 versus 0.327 ng/mg for DA, 0.114 versus 0.142 ng/mg for DOPAC, 0.424 versus 0.438 ng/mg for DOPAC/DA with p≤0.021 for DA, DOPAC and p>0.05 for DOPAC/DA, one-way ANOVA). In contrast, the defocus-only group showed similar levels of DA and its metabolites between eyes of the individual animals (DA: 0.288 versus 0.309 ng/mg, DOPAC: 0.115 versus 0126 ng/mg, DOPAC/DA: 0.407 versus 0.409 ng/mg; p>0.064: defocus versus defocus fellow, paired sample *t*-test). However, the defocused eyes showed a significant reduction in retinal DOPAC level but no significant changes in DA or DOPAC/DA levels when compared to the normal control group (Figure 1).

**Table 1:** Differences in refraction and concentration of retinal DA and DOPAC at 11 days of treatment (mean±SE, paired sample *t*-test).

**Groups**	**Refraction (Diopter)**		**Dopamine (ng/mg wet wt retina)**		**DOPAC(ng/mg wet wt retina)**		**DOPAC/DA**	
** **	**Experimental**	**Fellow**	**Experimental**	**Fellow**	**Experimental**	**Fellow**	**Experimental**	**Fellow**
Normal control (n=14)	6.29±0.44	6.52±0.57	0.327±0.013	0.329±0.012	0.142±0.008	0.152±0.017	0.438±0.020	0.459±0.041
** **	p=0.270		p=0.274		p=0.154		p=0.566	
FDM-only (n=26)	*2.92±0.36	6.16±0.28	*0.273±0.009	0.292±0.007	*0.114±0.005	0.134±0.007	*0.424±0.017	0.462±0.024
** **	p<0.001		p=0.042		p=0.001		p=0.019	
Defocus-only (n=23)	*2.26±0.20	6.44±0.20	0.288±0.011	0.309±0.010	0.115±0.004	0.126±0.005	0.407±0.014	0.409±0.008
** **	p<0.001		p=0.068		p=0.064		p=0.931	

Paired sample *t*-test was applied to the comparison between eyes of individual animals, and one-way ANOVA was applied to the comparison between FDM/defocused eye and normal control eyes.

### Intravitreous concentration of APO after subconjunctival injection

**Figure 2:**
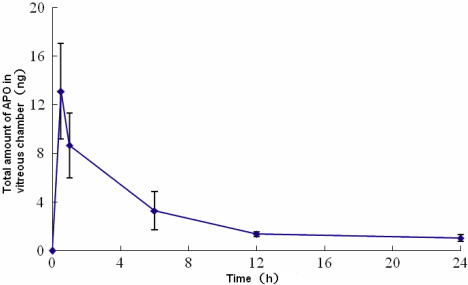
Time course of changes in total amount of APO in the vitreous chamber after subconjunctival injection of APO. The amount of APO in the vitreous chamber peaked 0.5 h or less after injection and decreased rapidly, reaching a plateau at 12 h and maintaining plateau levels at 24 h. The data at each time point was from cross-section measurements.

A single subconjunctival injection of 100 µg APO (100 µg in 100 µl: 1 mg/ml) produced a vitreous concentration of 87.51 ng/ml, 57.77 ng/ml, 21.84 ng/ml and 9.15 ng/ml in 0.5, 1, 6, and 12 h, respectively (Figure 2). The concentration at the 12-h time point was maintained for at least another 12 h. The total amount of drug in the vitreous chamber after subconjunctival injection of 100 µg APO was 13.13 ng, 8.67 ng, 3.28 ng, and 1.37 ng at 0.5, 1, 6, and 12 h after drug injection, respectively, based on an estimated volume of 0.15 ml for the guinea pig vitreous chamber (guinea pigs’ vitreous chamber length estimated as 0.36 cm, the vitreous chamber volume calculated according to volume formula: V=πr^3^). The amount of APO that reached the vitreous was only approximately 1/10^4^ of the amount that was injected subconjunctivally. Therefore, a subconjunctival injection of 100 µl of 2.5 ng/µl APO (the concentration and volume used in the present study) could produce 25 pg APO (4.37 nM) in the vitreous chamber 0.5 h after the injection with the amount maintained at approximately 8 pg (1.40 nM) within the first 6 h, followed by a constant amount of 4–5 pg (0.70–0.87 nM) until the next injection (given that the difference of diffusion index between different drug concentrations was neglected).

### Changes of refraction and axial components in different treatment groups

#### Baseline and normal control groups

**Figure 3:**
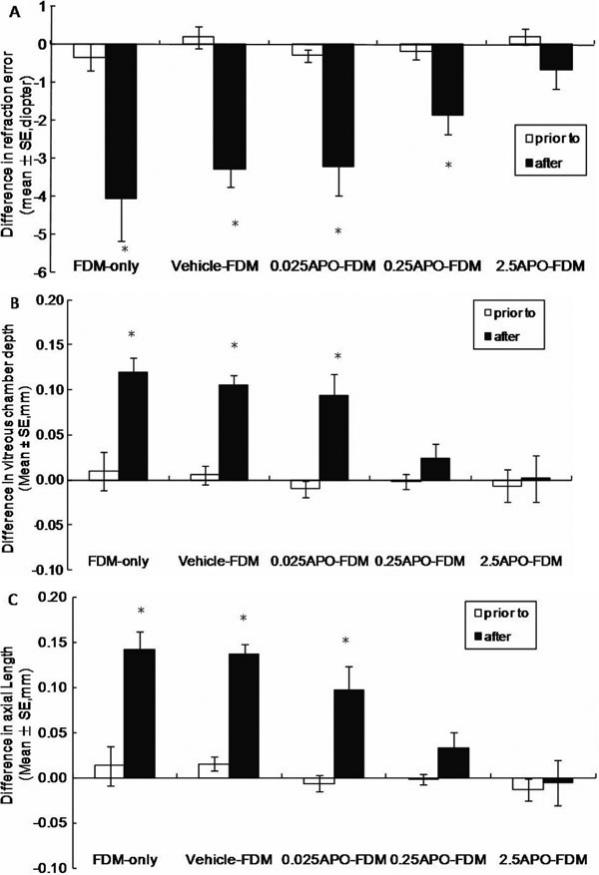
Biometric measurements in FDM, vehicle-FDM, and APO-FDM (0.025 to 250 ng/μl) groups before and at 11 days of FDM. **A**: Refraction; **B**: Vitreous length; **C**: Axial length. APO effectively blocked the development of FDM by inhibiting the excessive elongation of vitreous chamber in a dose-dependent pattern (* indicates a p<0.05 compared to the fellow eye, paired *t*-test).

**Figure 4:**
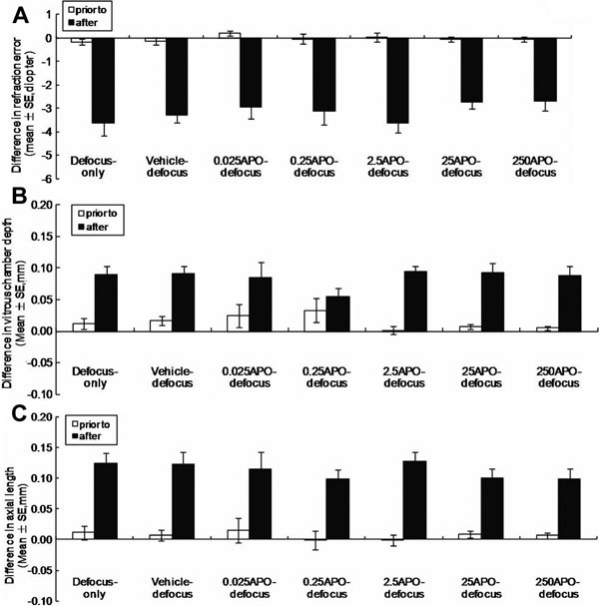
Biometric measurements in defocus-only, vehicle-defocus, and APO-defocus (0.025 to 250 ng/μl) groups before and at 11 days of hyperopic defocus. **A**: Refraction; **B**: Vitreous length; **C**: Axial length. At the concentrations examined, APO has no statistically significant effect on the development of defocus-induced myopia.

Prior to the experiment, there was no significant difference between eyes of individual animals in each group in refraction, corneal radius of curvature and various axial components (Figure 3 and Figure 4). There was also no significant difference in the right or left eyes between any two groups for any of the biometric results before the experiment (p>0.05, one-way ANOVA). Therefore, only results from the right eyes of the animals in the normal control group were used for comparison with the eyes from the other groups.

There was no significant difference between eyes of the same animals in each of the 3 control groups (normal control, vehicle-only and 2.5 ng/μl APO-only) in refraction, corneal radius of curvature and various axial components at day 11 of the experiment (p≥0.068, paired sample *t*-test).

#### FDM groups

There was no significant difference in any of the biometric results between the fellow eyes of each FDM group and the normal control eyes at 11 days (p≥0.314, right eyes in normal control vs fellow eyes in each FDM group, one-way ANOVA), indicating that the fellow eyes of all FDM groups can be used as a control to assess biometric changes in the deprived eyes. In all FDM groups, there was a myopic shift in refraction in the deprived eyes when compared to the fellow eyes. The largest myopic shift was observed in the FDM-only group (−4.06 D) and the vehicle-FDM group (−3.28 D). APO treatment inhibited the myopic shift in the deprived eyes compared to the fellow eyes (Figure 3A). In the 0.025 ng/µl APO-FDM group there was a shift of −3.20 D and in the 0.25 ng/µl APO-FDM group the shift decreased to −1.86 D when compared to the fellow eyes (p≤0.013, paired sample *t*-test). In the 2.5 ng/µl APO-FDM group, there was no difference in refraction between the deprived eyes and the fellow eyes (p=0.283, paired sample *t*-test), indicating that APO at this dose abolished the effect of form deprivation. In parallel with refractive changes, vitreous length of the deprived eyes increased significantly from day 0 to day 11 with a mean increase of 0.12 mm in the FDM-only group and 0.11 mm in the vehicle-FDM group (Figure 3B). Local APO treatment also slowed down vitreous lengthening of the deprived eyes in a concentration-dependent manner in the 0.25 ng/µl and 2.5 ng/µl APO-FDM groups since there was no difference between the deprived eyes and fellow eyes in these 2 groups (p≥0.170, paired sample *t*-test).

The corneal radius of curvature and lens thickness increased significantly from day 0 to day 11 in both eyes of individual animals in all the FDM groups (p<0.05: day 0 versus day 11 for all groups, independent *t*-test), except the cornea radius of curvature in the 250 ng APO-FDM group (p=0.079: day 0 versus day 11, independent *t*-test). There was no significant difference between the deprived and fellow eyes for these two parameters at any time point (p≥0.271 for corneal radius of curvature; p≥0.12 for lens thickness, paired sample *t*-test).

### Defocus groups

In all defocus-induced myopia groups, there were no significant differences in biometric results between the fellow eyes of all defocus groups and the right eyes of the normal control group at day 11 (p≥0.070: right eyes in normal control versus fellow eyes in each defocus group, one-way ANOVA). The defocused eyes in all defocus groups developed significant myopia at day 11 compared to the fellow eyes (p≤0.001, paired sample *t*-test). Consistent with the refractive changes, the vitreous length of the defocused eyes increased significantly from day 0 to day 11 in all the groups compared to the fellow eyes (p≤0.006, paired sample *t*-test). Corneal radius of curvature and lens thickness increased significantly over the 11-day in all defocus groups (p≤0.004: day 0 versus day 11, independent *t*-test). There was no significant difference of all the parameters between eyes of individual animals at any time point (p≥0.165, paired sample *t*-test) in all the groups.

## Discussion

This present study shows that degree of myopic shift with the associated increase in vitreous length is similar between FDM and defocus-induced myopia. However, the level of DA and DOPAC is lower in the deprived eyes but remains unchanged in the defocused eyes when compared to the fellow and normal control eyes, though a larger sample for defocus groups is needed to confirm the insignificant change in DA. These changes in DA synthesis and metabolism are consistent with previous studies on FDM [41,48] and defocus-induced myopia [41,48] in chickens, indicating that the dopaminergic system is involved in the development of FDM more actively than in the development of defocus-induced myopia. Given that the less involvement of the dopaminergic system in the defocus-induced myopia but similar biometric outcomes between the 2 models, another neurologic mechanism, such as the cholinergic system, may act as a “back-up” for the development of defocus-induced myopia to compensate the insufficient involvement of the dopaminergic system. This hypothesis is supported by an inhibitory effect of the combination of atropine and APO on defocus-induced myopia in chickens [16].

The dose-dependent inhibition of FDM by local APO injection is in agreement with previous findings that local injection of APO inhibits FDM in chickens and primates [27,28,42,62]. For example, subconjunctival injection of APO at 2.5 ng or intravitreal injection of APO at 5 pg to neonatal chickens (younger than 20 days) can reduce 50% of the FDM [27,42,63], while an increased dose to 250 ng APO can completely inhibit the FDM. The dosage curve of vitreous APO presented in this study indicates that the amount of APO was reduced significantly (by 4 orders) after the drug diffuses across the guinea pig sclera. The concentration range used in the experimental groups of this study (0.025 to 2.5 ng/µl) is 5 times lower than in chickens (0.125 to 12.5 ng/µl [42], ). Based on the dosage curve in this present study, subconjunctival injection of 250 ng APO produces a higher vitreous concentration than the E50 dosage (50% effective) used in the chickens (0.70–0.87 nM in guinea pig versus 0.056 nM in chicken [42] probably due to a more permeable mammalian sclera (not partially cartilage tissue as chicken sclera). The decrease in retinal DA biosynthesis in FDM supports a hypothesis that retinal DA regulates normal axial growth of the eye and a relatively hypodopaminergic state may contribute to the development of axial myopia [31,47]. It is noted that APO does not change the axial growth of the eye under normal visual environment. The different effects of DA agonist on eyes under FDM and normal visual development could be due to a higher affinity for exogenous DA in the deprived eyes compared to eyes exposed to a normal light cycle [44,64].

In contrast to FDM, APO does not significantly inhibit defocus-induced myopia even at a dose of 3 orders higher, probably due to a lower sensitivity of the defocused eyes to APO as the concentration of DA in these eyes is not reduced (compared to the fellow eyes), resulting in more saturated DA receptors in defocused-eyes. However, previous studies on chickens have shown that APO and 2-amino-6,7-dihydroxy-1,2,3,4-tetrahydronaphthanele hydrobromide (ADTN: another DA agonist) can significantly inhibit defocus-induced myopia in chickens [11,16]. It is noted that the negative power of the lenses used in these previous studies are much higher than −4.00 D and the animals used were much younger than those used in the present study (−15 D lens on 8 day old chickens) [16,27,42]. Therefore, the discrepancy in results of defocus-induced myopia between the present and previous studies may be due to differences in age of the animals (the younger the animals, the more susceptible to myopic development), compensation amplitude of the defocused eye and animal species used for experiments. More recent studies show that spiperone (D2 antagonist) completely inhibits the protective effect of temporal re-exposure of the deprived eye against FDM [62,65], but can partially inhibit defocus-induced myopia [62,65]. These results suggest an opposite role of dopamine agonists between FDM and defocus-induced myopia but again indicate that the involvement of doparminergic system is higher in FDM than in defocus-induced myopia.

Retinal DA release is sensitive to light exposure [38,64] and involved in visual stimulated signaling. The light transmittance and visual input in deprived eyes are weaker than in defocused eyes. This may explain the difference in retinal DA level observed in these two models (Figure 1). This notion is supported by previous findings as bright luminance and flickering can increase DA release and block FDM in chickens but only slows down or partially inhibits the development of defocus-induced myopia [7,18,40,46]. Form deprivation may obscure the dark-light cycle and therefore disrupts the circadian rhythm of ocular growth [41]. Restoring this circadian rhythm has been shown to inhibit FDM but not defocus-induced myopia [46]. In this present study, local injection of APO at 9:00 AM mimics the cycle of increased DA release that usually occurs during daytime and may help maintain the normal circadian rhythm of DA in the deprived eye. This could explain why local administration of APO can inhibit FDM but not defocus-induced myopia.

In summary, subconjuctival injection of APO can produce an effective concentration of intravitreous APO to attenuate myopic shift and axial elongation following form deprivation. However this inhibitory effect is less effective in defocus-induced myopia. Thus, DA signal appears to be critical in development of FDM but not necessarily involved in defocus-induced myopia, indicating that different neurochemical mechanisms may be involved in dopamine-mediated axial growth for these two visual manipulations.

## References

[1] Rohrer B, Iuvone PM, Stell WK (1995). Stimulation of dopaminergic amacrine cells by stroboscopic illumination or fibroblast growth factor (bFGF, FGF-2) injections: possible roles in prevention of form-deprivation myopia in the chick.. Brain Res.

[2] Wallman J, Winawer J (2004). Homeostasis of eye growth and the question of myopia.. Neuron.

[3] Rymer J, Wildsoet CF (2005). The role of the retinal pigment epithelium in eye growth regulation and myopia: a review.. Vis Neurosci.

[4] Rada JA, Wiechmann AF (2006). Melatonin receptors in chick ocular tissues: implications for a role of melatonin in ocular growth regulation.. Invest Ophthalmol Vis Sci.

[5] Mao J, Liu S, Qin W, Li F, Wu X, Tan Q (2010). Levodopa inhibits the development of form-deprivation myopia in guinea pigs.. Optom Vis Sci.

[6] Zhao J, Qu X, Qi Y, Zhou W, Liu X (2010). Study on retinal dopamine transporter in form deprivation myopia using the radiopharmaceutical tracer 99mTc-TRODAT-1.. Nucl Med Commun.

[7] Luft WA, Iuvone PM, Stell WK (2004). Spatial, temporal, and intensive determinants of dopamine release in the chick retina.. Vis Neurosci.

[8] Megaw PL, Boelen MG, Morgan IG, Boelen MK (2006). Diurnal patterns of dopamine release in chicken retina.. Neurochem Int.

[9] Siegwart JT, Norton TT (1993). Refractive and ocular changes in tree shrews raised with plus or minus lenses.. Invest Ophthalmol Vis Sci.

[10] Diether S, Schaeffel F (1997). Local changes in eye growth induced by imposed local refractive error despite active accommodation.. Vision Res.

[11] Ashby R, McCarthy CS, Maleszka R, Megaw P, Morgan IG (2007). A muscarinic cholinergic antagonist and a dopamine agonist rapidly increase ZENK mRNA expression in the form-deprived chicken retina.. Exp Eye Res.

[12] Jobling AI, Wan R, Gentle A, Bui BV, McBrien NA (2009). Retinal and choroidal TGF-beta in the tree shrew model of myopia: isoform expression, activation and effects on function.. Exp Eye Res.

[13] Ashby RS, Megaw PL, Morgan IG (2010). Changes in retinal alphaB-crystallin (cryab) RNA transcript levels during periods of altered ocular growth in chickens.. Exp Eye Res.

[14] Zhou X, Ye J, Willcox MD, Xie R, Jiang L, Lu R, Shi J, Bai Y, Qu J (2010). Changes in protein profiles of guinea pig sclera during development of form deprivation myopia and recovery.. Mol Vis.

[15] Gao H, Frost MR, Siegwart JT, Norton TT (2011). Patterns of mRNA and protein expression during minus-lens compensation and recovery in tree shrew sclera.. Mol Vis.

[16] Schmid KL, Wildsoet CF (2004). Inhibitory effects of apomorphine and atropine and their combination on myopia in chicks.. Optom Vis Sci.

[17] Diether S, Schaeffel F, Lambrou GN, Fritsch C, Trendelenburg AU (2007). Effects of intravitreally and intraperitoneally injected atropine on two types of experimental myopia in chicken.. Exp Eye Res.

[18] Ashby RS, Schaeffel F (2010). The Effect of Bright Light on Lens-Compensation in Chicks.. Invest Ophthalmol Vis Sci.

[19] Fang PC, Chung MY, Yu HJ, Wu PC (2010). Prevention of myopia onset with 0.025% atropine in premyopic children.. J Ocul Pharmacol Ther.

[20] Cottriall CL, McBrien NA (1996). The M1 muscarinic antagonist pirenzepine reduces myopia and eye enlargement in the tree shrew.. Invest Ophthalmol Vis Sci.

[21] Siatkowski RM, Cotter S, Miller JM, Scher CA, Crockett RS, Novack GD (2004). Safety and efficacy of 2% pirenzepine ophthalmic gel in children with myopia: a 1-year, multicenter, double-masked, placebo-controlled parallel study.. Arch Ophthalmol.

[22] Tan DT, Lam DS, Chua WH, Shu-Ping DF, Crockett RS (2005). One-year multicenter, double-masked, placebo-controlled, parallel safety and efficacy study of 2% pirenzepine ophthalmic gel in children with myopia.. Ophthalmology.

[23] Lee JJ, Fang PC, Yang IH, Chen CH, Lin PW, Lin SA, Luo HK, Wu PC (2006). Prevention of myopia progression with 0.05% atropine solution.. J Ocul Pharmacol Ther.

[24] Siatkowski RM, Cotter SA, Crockett RS, Miller JM, Novack GD, Zadnik K (2008). Two-year multicenter, randomized, double-masked, placebo-controlled, parallel safety and efficacy study of 2% pirenzepine ophthalmic gel in children with myopia.. J AAPOS.

[25] Kennedy RH, Dyer JA, Kennedy MA, Parulkar S, Kurland LT, Herman DC, Mclntire D, Jacobs D, Luepker RV (2000). Reducing the progression of myopia with atropine: a long term cohort study of Olmsted County students.. Binocul Vis Strabismus Q.

[26] Tong L, Huang XL, Koh AL, Zhang X, Tan DT, Chua WH (2009). Atropine for the treatment of childhood myopia: effect on myopia progression after cessation of atropine.. Ophthalmology.

[27] Stone RA, Lin T, Laties AM, Iuvone PM (1989). Retinal dopamine and form-deprivation myopia.. Proc Natl Acad Sci USA.

[28] Iuvone PM, Tigges M, Stone RA, Lambert S, Laties AM (1991). Effects of apomorphine, a dopamine receptor agonist, on ocular refraction and axial elongation in a primate model of myopia.. Invest Ophthalmol Vis Sci.

[29] Pezzoli G, Zini M (2010). Levodopa in Parkinson's disease: from the past to the future.. Expert Opin Pharmacother.

[30] Gao Q, Liu Q, Ma P, Zhong X, Wu J, Ge J (2006). Effects of direct intravitreal dopamine injections on the development of lid-suture induced myopia in rabbits.. Graefes Arch Clin Exp Ophthalmol.

[31] Iuvone PM, Tigges M, Stone RA, Lambert S, Laties AM (1991). Effects of apomorphine, a dopamine receptor agonist, on ocular refraction, and axial elongation in a primate model of myopia.. Invest Ophthalmol Vis Sci.

[32] Nickla DL, Totonelly K, Dhillon B (2010). Dopaminergic agonists that result in ocular growth inhibition also elicit transient increases in choroidal thickness in chicks.. Exp Eye Res.

[33] Zhu X, Park TW, Winawer J, Wallman J (2005). In a matter of minutes, the eye can know which way to grow.. Invest Ophthalmol Vis Sci.

[34] Howlett MH, McFadden SA (2006). Form-deprivation myopia in the guinea pig (Cavia porcellus).. Vision Res.

[35] Zhou X, Lu F, Xie R, Jiang L, Wen J, Li Y, Shi J, He T, Qu J (2007). Recovery from axial myopia induced by a monocularly deprived facemask in adolescent (7-week-old) guinea pigs.. Vision Res.

[36] Chen YP, Prashar A, Hocking PM, Erichsen JT, To CH, Schaeffel F, Guggenheim JA (2010). Sex, eye size, and the rate of myopic eye growth due to form deprivation in outbred white leghorn chickens.. Invest Ophthalmol Vis Sci.

[37] Wildsoet C, Wallman J (1995). Choroidal and scleral mechanisms of compensation for spectacle lenses in chicks.. Vision Res.

[38] Umino O, Lee Y, Dowling JE (1991). Effects of light stimuli on the release of dopamine from interplexiform cells in the white perch retina.. Vis Neurosci.

[39] Dong CJ, McReynolds JS (1992). Comparison of the effects of flickering and steady light on dopamine release and horizontal cell coupling in the mudpuppy retina.. J Neurophysiol.

[40] Schwahn HN, Schaeffel F (1997). Flicker parameters are different for suppression of myopia and hyperopia.. Vision Res.

[41] Schaeffel F, Bartmann M, Hagel G, Zrenner E (1995). Studies on the role of the retinal dopamine/melatonin system in experimental refractive errors in chickens.. Vision Res.

[42] Rohrer B, Spira AW, Stell WK (1993). Apomorphine blocks form-deprivation myopia in chickens by a dopamine D2-receptor mechanism acting in retina or pigmented epithelium.. Vis Neurosci.

[43] Ohngemach S, Hagel G, Schaeffel F (1997). Concentrations of biogenic amines in fundal layers in chickens with normal visual experience, deprivation, and after reserpine application.. Vis Neurosci.

[44] Ohngemach S, Feldkaemper M, Schaeffel F (2001). Pineal control of the dopamine D2-receptor gene and dopamine release in the retina of the chicken and their possible relation to growth rhythms of the eye.. J Pineal Res.

[45] Fujikado T, Kawasaki Y, Suzuki A, Ohmi G, Tano Y (1997). Retinal function with lens-induced myopia compared with form-deprivation myopia in chicks.. Graefes Arch Clin Exp Ophthalmol.

[46] Kee CS, Marzani D, Wallman J (2001). Differences in time course and visual requirements of ocular responses to lenses and diffusers.. Invest Ophthalmol Vis Sci.

[47] Iuvone PM, Tigges M, Fernandes A, Tigges J (1989). Dopamine synthesis and metabolism in rhesus monkey retina: development, aging, and the effects of monocular visual deprivation.. Vis Neurosci.

[48] Pendrak K, Nguyen T, Lin T, Capehart C, Zhu X, Stone RA (1997). Retinal dopamine in the recovery from experimental myopia.. Curr Eye Res.

[49] Guo SS, Sivak JG, Callender MG, Diehl-Jones B (1995). Retinal dopamine and lens-induced refractive errors in chicks.. Curr Eye Res.

[50] Bartmann M, Schaeffel F, Hagel G, Zrenner E (1994). Constant light affects retinal dopamine levels and blocks deprivation myopia but not lens-induced refractive errors in chickens.. Vis Neurosci.

[51] Phillips JR, Khalaj M, McBrien NA (2000). Induced myopia associated with increased scleral creep in chick and tree shrew eyes.. Invest Ophthalmol Vis Sci.

[52] Smith EL, Hung LF, Kee CS, Qiao Y (2002). Effects of brief periods of unrestricted vision on the development of form-deprivation myopia in monkeys.. Invest Ophthalmol Vis Sci.

[53] McFadden SA, Howlett MH, Mertz JR (2004). Retinoic acid signals the direction of ocular elongation in the guinea pig eye.. Vision Res.

[54] Lu F, Zhou X, Zhao H, Wang R, Jia D, Jiang L, Xie R, Qu J (2006). Axial myopia induced by a monocularly-deprived facemask in guinea pigs: A non-invasive and effective model.. Exp Eye Res.

[55] Howlett MH, McFadden SA (2007). Emmetropization and schematic eye models in developing pigmented guinea pigs.. Vision Res.

[56] Lu F, Zhou X, Jiang L, Fu Y, Lai X, Xie R, Qu J (2009). Axial myopia induced by hyperopic defocus in guinea pigs: A detailed assessment on susceptibility and recovery.. Exp Eye Res.

[57] Qiao-Grider Y, Hung LF, Kee CS, Ramamirtham R, Smith EL (2004). Recovery from form-deprivation myopia in rhesus monkeys.. Invest Ophthalmol Vis Sci.

[58] Troilo D, Nickla DL (2005). The response to visual form deprivation differs with age in marmosets.. Invest Ophthalmol Vis Sci.

[59] Smith EL, Huang J, Hung LF, Blasdel TL, Humbird TL, Bockhorst KH (2009). Hemiretinal form deprivation: evidence for local control of eye growth and refractive development in infant monkeys.. Invest Ophthalmol Vis Sci.

[60] Weber B, Schlicker E, Sokoloff P, Stark H (2001). Identification of the dopamine autoreceptor in the guinea-pig retina as D(2) receptor using novel subtype-selective antagonists.. Br J Pharmacol.

[61] Baranyi M, Milusheva E, Vizi ES, Sperlagh B (2006). Chromatographic analysis of dopamine metabolism in a Parkinsonian model.. J Chromatogr A.

[62] McCarthy CS, Megaw P, Devadas M, Morgan IG (2007). Dopaminergic agents affect the ability of brief periods of normal vision to prevent form-deprivation myopia.. Exp Eye Res.

[63] Stone RA, Lin T, Iuvone PM, Laties AM (1990). Postnatal control of ocular growth: dopaminergic mechanisms.. Ciba Found Symp.

[64] Witkovsky P (2004). Dopamine and retinal function.. Doc Ophthalmol.

[65] Nickla DL, Totonelly K, Dhillon B (2010). Dopaminergic agonists that result in ocular growth inhibition also elicit transient increases in choroidal thickness in chicks.. Exp Eye Res.

